# Reduced serum LEFTY2 and disease-dependent biomarker associations in Alzheimer’s disease: an exploratory study

**DOI:** 10.3389/fnagi.2026.1955495

**Published:** 2026-10-06

**Authors:** Wei Wuli, Raymond Y. Lo, Horng-Jyh Harn, Tzyy-Wen Chiou, Yue-Xuan Xu, Yuncin Luo, Ivan Wijaya, Shinn-Zong Lin

**Affiliations:** 1 Bioinnovation Center, Hualien Tzu Chi Hospital, Hualien, Taiwan; 2 Center for Dementia Care, St. Mary’s Hospital, Taitung, Taiwan; 3 Department of Biochemistry and Molecular Medicine, National Dong Hwa University, Hualien, Taiwan; 4 Department of Neurology, Hualien Tzu Chi Hospital, Hualien, Taiwan; 5 Department of Pathology, Hualien Tzu Chi Hospital, Hualien, Taiwan; 6 Ph.D. Program of Interdisciplinary Medicine, National Yang Ming Chiao Tung University, Taipei, Taiwan; 7 Department of Neurosurgery, Hualien Tzu Chi Hospital, Hualien, Taiwan

**Keywords:** Alzheimer’s disease, Aβ1-42, blood-based biomarkers, LEFTY2, pTau181, TGF-β1

## Abstract

Reliable blood-based biomarkers are increasingly needed for the detection and monitoring of Alzheimer’s disease (AD). Left–right determination factor 2 (LEFTY2) is a secreted protein of the transforming growth factor-β (TGF-β) superfamily. Evidence from AD-related neuronal models suggests that LEFTY2 promotes neuronal survival, attenuates amyloid- and tau-related pathology, and suppresses inflammatory responses, highlighting its potential relevance to AD. However, its clinical significance and relationship with AD-related blood biomarkers remain unclear. In this study, we investigated the association between serum LEFTY2 and and biomarkers associated with amyloid pathology, tau pathology, neuroinflammation, and neurodegeneration in patients with AD and age-matched cognitively normal (CN) controls. Biomarker levels were quantified using enzyme-linked immunosorbent assays. Serum LEFTY2 levels were significantly lower in AD patients than in cognitively normal controls, whereas Amyloid-beta 1–40 (Aβ1-40), phosphorylated tau (pTau) 181 and Neurofilament light chain (NF-L) levels were significantly elevated. Correlation analyses revealed a positive association between LEFTY2 and Aβ1-42 in AD patients, while correlations with biomarkers of tau pathology, inflammation, and neurodegeneration were less consistent. In cognitively normal controls, LEFTY2 was negatively correlated with total tau and positively correlated with TGF-β1. Collectively, these findings indicate that LEFTY2 exhibits distinct associations with AD-related biomarkers in a disease-dependent manner. These exploratory findings provide new insights into the relationships among circulating AD biomarkers and support further investigation of LEFTY2 as a novel candidate blood-based marker in larger prospective cohorts.

## 1 Introduction

Alzheimer’s disease (AD) is a multifactorial neurodegenerative disorder characterized by amyloid-beta (Aβ) deposition, tau pathology, neurodegeneration, and neuroinflammation, all of which contribute to disease progression through complex biological processes (Jack Jr et al., 2024; Heneka et al., 2025). This complexity has driven increasing interest in identifying reliable biomarkers that not only facilitate early detection and disease monitoring but also improve our understanding of the biological mechanisms underlying AD (Therriault et al., 2024).

In recent years, blood-based biomarkers have emerged as promising tools for the non-invasive detection and monitoring of AD (Mielke and Fowler, 2024). Circulating biomarkers, including Aβ peptides, phosphorylated tau species, and inflammatory mediators, reflect distinct aspects of amyloid pathology, tau pathology, neurodegeneration, and neuroinflammation (Dark et al., 2024). While these biomarkers have substantially improved the biological diagnosis of AD, increasing attention has shifted from evaluating individual biomarkers toward examining their associations across different pathological domains. Understanding these associations may provide additional insight into the biological processes underlying AD. However, the upstream factors that influence these biomarker associations remain poorly understood.

LEFTY2, a member of the transforming growth factor-β (TGF-β) superfamily, is best known for its role in embryonic development and left–right axis formation (Wu and Hill, 2009). Beyond development, dysregulation of TGF-β signaling has been implicated in several pathological processes involved in AD, including amyloid deposition, tau pathology, neuroinflammation, and neurodegeneration. As an endogenous modulator of TGF-β signaling, LEFTY2 may therefore influence multiple aspects of AD pathology. In our previous study, we identified LEFTY2 as a bioactive factor secreted by adipose-derived stem cells following co-culture with trisomy 21-derived neurons exhibiting AD-like pathology. Recombinant LEFTY2 promoted neuronal survival while reducing amyloid-β accumulation, tau phosphorylation, APOE4 expression, and inflammatory signaling, suggesting a potential role for LEFTY2 in modulating AD-related pathological changes (Wuli et al., 2025).

Despite these promising experimental findings, the clinical significance of circulating LEFTY2 in patients with AD remains unknown. In particular, whether serum LEFTY2 is associated with established blood biomarkers reflecting amyloid pathology, tau pathology, neurodegeneration, and neuroinflammation have not been systematically investigated. Addressing this question may improve insight into the biological relevance of LEFTY2 in AD and generate new hypotheses regarding its potential involvement in AD-related pathophysiology.

Therefore, in this exploratory study, we quantified serum LEFTY2 together with established AD-related blood biomarkers in patients with AD and age-matched cognitively normal controls. We examined the associations between serum LEFTY2 and biomarkers reflecting amyloid pathology, tau pathology, neurodegeneration, and neuroinflammation across disease groups and age strata. We hypothesized that serum LEFTY2 is differentially associated with biomarkers representing distinct pathological processes in AD, providing new insight into its potential biological role and clinical relevance.

## 2 Materials and methods

### 2.1 AD patients diagnosis and recruitment

AD patients were recruited from Hualien Tzu Chi Hospital, Taiwan (*n* = 15). Diagnosis of AD was established through comprehensive clinical evaluation by a board-certified neurologist based on presenting clinical symptoms, such as progressive memory impairment, short-term retrieval deficits, or word-finding difficulties. Cognitive status and disease severity were characterized using the Mini-Mental State Examination (MMSE) and the Clinical Dementia Rating (CDR) scale, encompassing CDR 0.5 (very mild), CDR 1.0 (mild dementia), and CDR 2.0 (moderate dementia).

Structural neuroimaging via brain magnetic resonance imaging (MRI) was available for all participants to characterize supportive features of neurodegeneration, including generalized cerebral and temporal atrophy, and to assess potential structural or vascular abnormalities. Mild-to-moderate cerebral small-vessel disease was noted in 14 patients. Molecular and fluid biomarkers, including amyloid/tau PET and CSF Aβ/tau measurements, were not systematically available across the cohort. Detailed subject-level clinical characteristics, neurocognitive scores, and individual structural MRI findings are summarized in Table 1.

**Table 1:** Clinical, cognitive, and MRI characteristics of the AD cohort.

ID	Age/sex	Presenting symptoms	MMSE	CDR	MRI impression	Exclusion criteria for non-AD dementia
01	74/F	Retrieval deficits, word-finding difficulty	17.00	0.50	Generalized cortical atrophy, mild temporal atrophy, mild periventricular small-vessel disease	No acute infarction or mass lesion; small-vessel disease present
02	87/F	Forgetfulness, visual–spatial impairment (cannot copy cube/interpret picture)	16.00	1.00	Generalized cortical atrophy, proportionate ventricular expansion, periventricular small-vessel disease	Microvascular changes present; extrapyramidal/DLB features excluded clinically
03	84/F	Short-term memory decline, general cognitive forgetfulness	19.00	1.00	Senile brain atrophy, CSF space widening, mild small-vessel disease (Fazekas 1)	No intracranial hemorrhage or structural non-AD lesion
04	81/M	Memory decline, retrieval deficits, safety/forgetting issues (tap water/lights)	20.00	0.50	Senile brain atrophy, proportional ventricular enlargement, mild small-vessel disease (Fazekas 1)	Mild microvascular changes; no acute stroke or mass lesion
05	88/F	Delusions, self-talking, severe retrieval/naming loss, stove safety issues	18.00	1.00	Cortical atrophy, mild hydrocephalus with transependymal CSF shift, 3.5 mm ICA aneurysm	Mild hydrocephalus & vascular changes noted; clinical correlation required
06	77/M	Short-term memory loss, temporal disorientation, attention deficit	19.00	2.00	Large left temporoparietal-occipital AVM with perifocal gliosis & tissue loss, generalized atrophy	No clinical stroke history; large AVM present and may contribute to impairment
07	70 / M	Forgetfulness, misplacing items, disorientation to year, picture misinterpretation	20.00	0.50	Moderate small-vessel disease (Fazekas 2), two old lacunar infarcts (left corona radiata)	Mixed vascular/AD pathology suspected due to chronic lacunes & arterial stenosis
08	71/F	Short-term memory decline, mild atrophy on imaging, depression/anxiety	26.00	0.50	Small T2-FLAIR hyperintense white matter nodules (small-vessel ischemia), chronic sinusitis	No mass lesion, hydrocephalus, or acute infarction identified
09	77/F	Progressive dementia, memory loss	18.00	1.00	Senile brain atrophy, small-vessel ischemic disease, intracranial atherosclerosis	No intracranial tumor, AVM, or acute stroke explaining dementia
10	75/M	Forgetfulness, reduced time orientation, cortical atrophy	20.00	1.00	Senile brain atrophy, old right parietal lacunar infarct, focal hemosiderin deposits	Focal chronic microvascular lesions present; no acute structural pathology
11	80/M	Repetitive questioning, short-term memory decline	22.00	1.00	Senile cortical atrophy, periventricular small-vessel ischemic disease	No definite neuroimaging evidence of non-AD primary etiology
12	64/M	Memory decline and impairment, unable to learn new material; biomarker-supported (positive [18F]florbetaben Amyloid PET and AD-pattern FDG-PET)	7.00	2.00	Generalized senile atrophy, moderate small-vessel disease (Fazekas 2), mild R-MCA stenosis	Microvascular disease & arterial stenosis noted
13	81/F	Cognitive decline, short-term memory impairment, attention deficit	8.00	2.00	Generalized senile brain atrophy, moderate small-vessel disease (Fazekas grade 2), mild right MCA-M1 stenosis, and right frontal superficial siderosis	Severe cognitive impairment; microvascular, stenotic, and siderotic changes present
14	76/F	Forgetfulness, repetitive checking behavior	22.00	0.50	Senile cortical atrophy, periventricular small-vessel ischemia, intracranial atherosclerosis	Microvascular/atherosclerotic changes present; no acute infarction or mass lesion
15	74/F	Progressive memory decline	11.00	1.00	Mild small-vessel disease (Fazekas 1), focal vertebral artery stenosis, two small ICA/ACA aneurysms	Mild microvascular disease & vascular stenosis; no acute hemorrhage/infarct

ACA, Anterior Cerebral Artery; AD, Alzheimer’s Disease; AVM, Arteriovenous Malformation; CDR, Clinical Dementia Rating; CSF, Cerebrospinal Fluid; DLB, Dementia with Lewy Bodies; F, Female; FDG-PET, [^18^F]Fluorodeoxyglucose Positron Emission Tomography; FLAIR, Fluid-Attenuated Inversion Recovery; ICA, Internal Carotid Artery; M, Male; MCA, Middle Cerebral Artery; MMSE, Mini-Mental State Examination; MRI, Magnetic Resonance Imaging; PET, Positron Emission Tomography.

### 2.2 Mini-mental state examination

Global cognitive function was evaluated using the Mini-Mental State Examination (MMSE), a standardized 30-point cognitive screening instrument widely used for assessment of cognitive impairment and dementia severity. The MMSE evaluates multiple cognitive domains, including orientation, registration, attention and calculation, recall, language, and visuospatial ability. The assessment was performed by trained clinical personnel in a quiet environment, and participants were instructed to complete all tasks according to standardized MMSE administration procedures.

The total MMSE score ranges from 0 to 30 points, with higher scores indicating better cognitive performance. For descriptive interpretation of cognitive performance, scores of 24–30 were considered within the generally preserved range, whereas scores ≤23 were considered indicative of cognitive impairment. MMSE scores were used to characterize cognitive performance and were interpreted in conjunction with the clinical diagnosis.

### 2.3 Serum isolation

Human peripheral blood samples were obtained from study participants following informed consent and approval by the Hualien Tzu Chi Hospital Research Ethics Committee (IRB112-222-B). Blood was collected by venipuncture into serum separator tubes and allowed to clot at room temperature for 30–60 min. Samples were centrifuged at 1,500 × g for 10 min to separate serum from cellular components. The serum fraction was carefully aspirated and transferred to sterile tubes, followed by a second centrifugation at 3,000 × g for 10 min to remove residual cells and debris. Serum samples were aliquoted and stored at −80 °C until use for biomarker analysis.

### 2.4 Enzyme-linked immunosorbent assay

ELISAs were performed across human serum samples using commercially available ELISA kits: Human Lefty-A ELISA (Invitrogen, EH298RB), Human Aβ42 ELISA Kit (Invitrogen, KHB3441), Human Aβ40 ELISA Kit (Invitrogen, KHB3481), Human TAU [pT231] Elisa Kit (Invitrogen, KHB8051), Human TAU [pT181] Elisa Kit (Invitrogen, KHO0631), Human TAU (total) ELISA Kit (Invitrogen, KHB0041), Human MIF ELISA (Invitrogen, EHMIF), Human TREM-2 ELISA (Invitrogen, EH464RB), Human TGF beta 1 ELISA Kit (Invitrogen, BMS249-4), and Human NF-L ELISA Kit (Invitrogen, BMS2362) following the manufacturer’s instructions. The optical density of the developed colorimetric reaction was measured at 450 nm using Thermo Scientific Multiskan GO (Thermo Fisher Scientific, 51,119,200), and data acquisition was performed using SkanIt Software 4.1 for Microplate Readers. All experiments were conducted in duplicate for human serum and triplicates for standard. Sample concentrations were determined by interpolating background-corrected optical density values from four-parameter logistic (4PL) regression curve generated from the standard series and applying the corresponding sample dilution factor. The analytical limit of detection (LOD) and limit of quantification (LOQ) were determined from the blank optical density values as follows:

$${\mathrm{LOD}}_{\mathrm{OD}}={\text{Mean}}_{\text{blank}}+(3\times {\mathrm{SD}}_{\text{blank}})$$

$${\mathrm{LOQ}}_{\mathrm{OD}}={\text{Mean}}_{\text{blank}}+(10\times {\mathrm{SD}}_{\text{blank}})$$

The corresponding LOD and LOQ concentration limits were obtained by interpolation from the 4PL standard curve and are summarized for each assay in Table 2. The lower limit of quantification (LLOQ) was defined as the concentration of the lowest non-zero standard within the quantifiable range of each assay. Samples with concentrations below the LLOQ were considered below the quantifiable range. For graphical representation and statistical analysis, concentrations below the LLOQ were imputed as:

$${\text{LLOQ}}_{\mathrm{OD}}=\mathrm{LOD}/\surd 2$$

**Table 2:** LOD and LOQ of serum biomarker assays.

Serum biomarker	Limit of detection (LOD)	Limit of quantification (LOQ)	Cognitively normal below LOD, n/15 (%)	AD below LOD, n/15 (%)
LEFTY2	~ 1.4 pg./mL	~ 4.5 pg./mL	0/15 (0.00%)	4/15 (26.67%)
Aβ1-42	~ 2.43 pg./mL	~ 2.62 pg./mL	0/15 (0.00%)	3/15 (20.00%)
Aβ1-40	~ 2.38 pg./mL	~ 2.87 pg./mL	0/15 (0.00%)	0/15 (0.00%)
pTau231	~ 0.07 U/mL	~ 0.13 U/mL	15/15 (100.00%)	7/15 (46.67%)
pTau181	~ 2.83 pg./mL	~ 2.83 pg./mL	15/15 (100.00%)	5/15 (33.33%)
Total Tau	~ 24.23 pg./mL	~ 29.35 pg./mL	1/15 (6.67%)	0/15 (0.00%)
MIF	~ 1.14 pg./mL	~ 17.21 pg./mL	8/15 (53.33%)	6/15 (40.00%)
TREM2	~ 3.74 pg./mL	~ 17.20 pg./mL	6/15 (40.00%)	7/15 (46.67%)
TGF-β1	~ 6.25 pg./mL	~ 15.31 pg./mL	0/15 (0.00%)	0/15 (0.00%)
NF-L	~ 0.37 pg./mL	~ 0.37 pg./mL	8/15 (53.33%)	2/15 (13.33%)

### 2.5 Statistical analysis

Normality was assessed using the Shapiro–Wilk test, with *p* < 0.05 indicating a significant deviation from normality, and homogeneity of variance was evaluated using Levene’s test. Group differences were evaluated using unpaired Student’s t-tests for normally distributed variables or Mann–Whitney U tests for non-normally distributed variables. Data are presented as mean ± standard error of the mean (SEM) alongside individual data points, with each point represents the average of duplicate ELISA measurements from one biological subject.

To account for multiple comparisons across the multi-biomarker panel and control the false discovery rate, raw two-tailed *p*-values were adjusted using the Benjamini-Hochberg False Discovery Rate (FDR) procedure. A total of 26 primary biomarker tests were performed, comprising 10 between-group biomarker comparisons and 16 LEFTY2–biomarker correlation analyses (eight correlations evaluated separately in the AD and cognitively normal groups). FDR correction was applied separately within the between-group comparison and correlation families, with statistical significance defined as q < 0.05. For all statistical charts, significance is denoted using a maximum two-tier asterisk convention: *p* < 0.05 (or q < 0.05) is indicated by a single asterisk (*), while *p* < 0.01 (or q < 0.01) is indicated by two asterisks (**). No more than two asterisks are displayed, irrespective of smaller p- or q-values.

Relationships between biomarker pairs were visualized using scatterplots of absolute protein concentrations (pg/mL) and quantified using two-tailed Spearman correlation coefficients (ρ). The sign of the correlation coefficient indicates the direction of the association, with positive values (ρ > 0) indicating a positive association and negative values (ρ < 0) indicating an inverse association. Correlation coefficients and group differences are reported with corresponding 95% confidence intervals (95% CIs).

To assess the robustness of the findings and determine whether the observed results were disproportionately influenced by individual observations, a formal sensitivity analysis was performed using the robust regression and outlier removal (ROUT) method with a false discovery rate parameter of Q = 1%. Outlier detection was conducted based on serum LEFTY2 concentrations across the entire study cohort (*n* = 30). The ROUT procedure identified three observations in the AD group and three observations in the cognitively normal group. These observations were excluded only for the sensitivity analysis, resulting in 12 participants per group (*n* = 24 total), while all participants were retained in the primary analyses.

## 3 Results

### 3.1 Serum LEFTY2 is decreased in AD patients

To investigate alterations in circulating AD-related biomarkers, serum samples from cognitively normal controls and AD patients (Table 3) were analyzed by ELISA. Biomarker concentrations were quantified using duplicate measurements for each individual and compared between groups. No significant difference in age distribution was observed between cognitively normal controls and AD patients, consistent with appropriate age matching between cohorts (cognitively normal = 74.20 ± 1.18 years, AD = 77.33 ± 1.68 years; *p* = 0.14; Figure 1A).

**Table 3:** Participant characteristics.

Participant characteristics	All (*n* = 30)	Cognitively normal (*n* = 15)	AD (*n* = 15)
Age, years (SD)	77.77 (5.75)	74.73 (1.48)	76.91 (1.57)
Females, n (%)	24.00 (80.00%)	14.00 (93.33%)	9.00 (60.00%)
Median MMSE (IQR)	28.00 (11.00)	30.00 (0.00)	19.00 (3.50)
Disease duration, years (SD)	2.87 (3.66)	0.00 (0.00)	5.73 (3.17)

AD, Alzheimer’s Disease; MMSE, Mini-Mental State Examination; SD, Standard Deviation; IQR, Interquartile Range.

**Figure 1:**
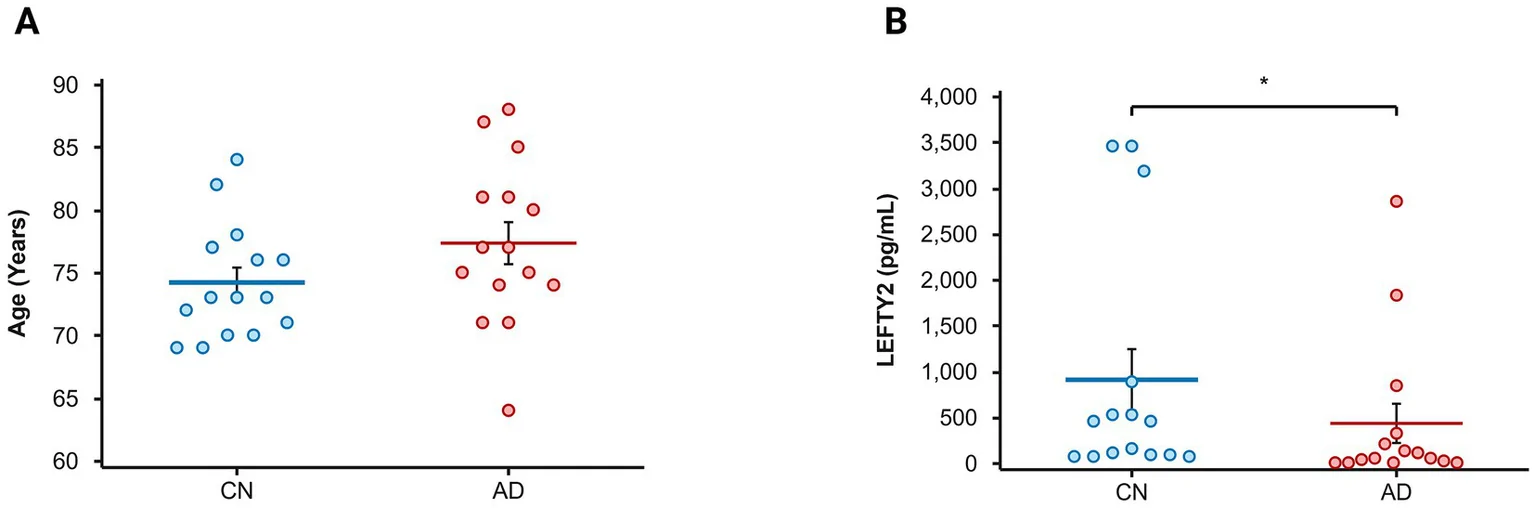
Age distribution and serum LEFTY2 levels in cognitively normal controls and AD patients. **(A)** Dot plots showing age distribution between cognitively normal controls and Alzheimer’s disease (AD) patients. No significant difference in age distribution was observed between groups, confirming appropriate age matching. **(B)** Scatter plots showing serum LEFTY2 concentrations in cognitively normal (CN) controls and AD patients. Serum LEFTY2 levels were significantly lower in AD patients compared with CN. Each data point represents the mean of duplicate measurements from an individual subject, while horizontal lines indicate group means ± SEM. Statistical significance was determined using unpaired Student’s t-test for age comparison and the Mann–Whitney U test for LEFTY2 and (*n* = 15 for each group).

Serum LEFTY2 levels were significantly lower in AD patients compared with cognitively normal controls, with mean values in the AD group approaching near-undetectable levels (cognitively normal = 905.73 ± 334.62 pg./mL, 95% CI: 249.86 to 1561.59; AD = 430.58 ± 213.68 pg./mL, 95% CI: 11.76 to 849.39; *p* = 0.03; q = 0.04; Figure 1B).

Due to extreme values observed within the cohort, we assessed whether these primary findings were driven by individual outliers. ROUT method identified 6 statistical outliers in total (3 in the cognitively normal control group and 3 in the AD group), resulting in a sensitivity-analysis dataset of *n* = 12 participants per group. Diagnostic group comparison for LEFTY2 was repeated using the sensitivity-analysis dataset (Supplementary Figure 1). Two-tailed Spearman correlation analyses between LEFTY2 and the other biomarkers were also repeated separately in the AD group (Supplementary Figure 2) and cognitively normal control group (Supplementary Figure 3). For these correlation analyses, listwise deletion was applied such that participants identified as LEFTY2 outliers were excluded together with their corresponding paired biomarker measurements.

Although partial overlap among several low-value samples was observed, these findings suggest a reduction in serum LEFTY2 levels in AD patients, providing a rationale for subsequent analyses examining its relationship with established AD biomarkers.

### 3.2 Serum LEFTY2 is significantly associated with Aβ1-42 in AD patients

To explore established Alzheimer’s disease-related biomarker alterations and their potential relationship with LEFTY2, serum Aβ isoforms were next analyzed. Aβ1-42 was significantly higher in cognitively normal controls than in AD patients (cognitively normal = 15.63 ± 3.46 pg./mL, 95% CI: 8.84 to 22.41; AD = 7.26 ± 1.27 pg./mL, 95% CI: 4.77 to 9.76; *p* = 0.03; q = 0.04; Figure 2A). This pattern may suggest reduced levels of soluble peripheral Aβ1-42 in AD patients. In contrast, serum Aβ1-40 levels were significantly elevated in AD patients (cognitively normal = 203.19 ± 18.67 pg./mL, 95% CI: 166.59 to 239.79; AD = 371.18 ± 56.71 pg./mL, 95% CI: 260.03 to 482.33; *p* = 0.02; q = 0.04, Figure 2B). Diagnostic group comparisons for Aβ1-42 and Aβ1-40 were also repeated using the sensitivity-analysis dataset (*n* = 12 participants per group; Supplementary Figure 1). Accordingly, the Aβ1-42/Aβ1-40 ratio was reduced in AD patients (cognitively normal = 0.08 ± 0.02, 95% CI: 0.04 to 0.12; AD = 0.02 ± 0.005, 95% CI: 0.02 to 0.03; *p* < 0.01; q = 0.04; Figure 2C).

**Figure 2:**
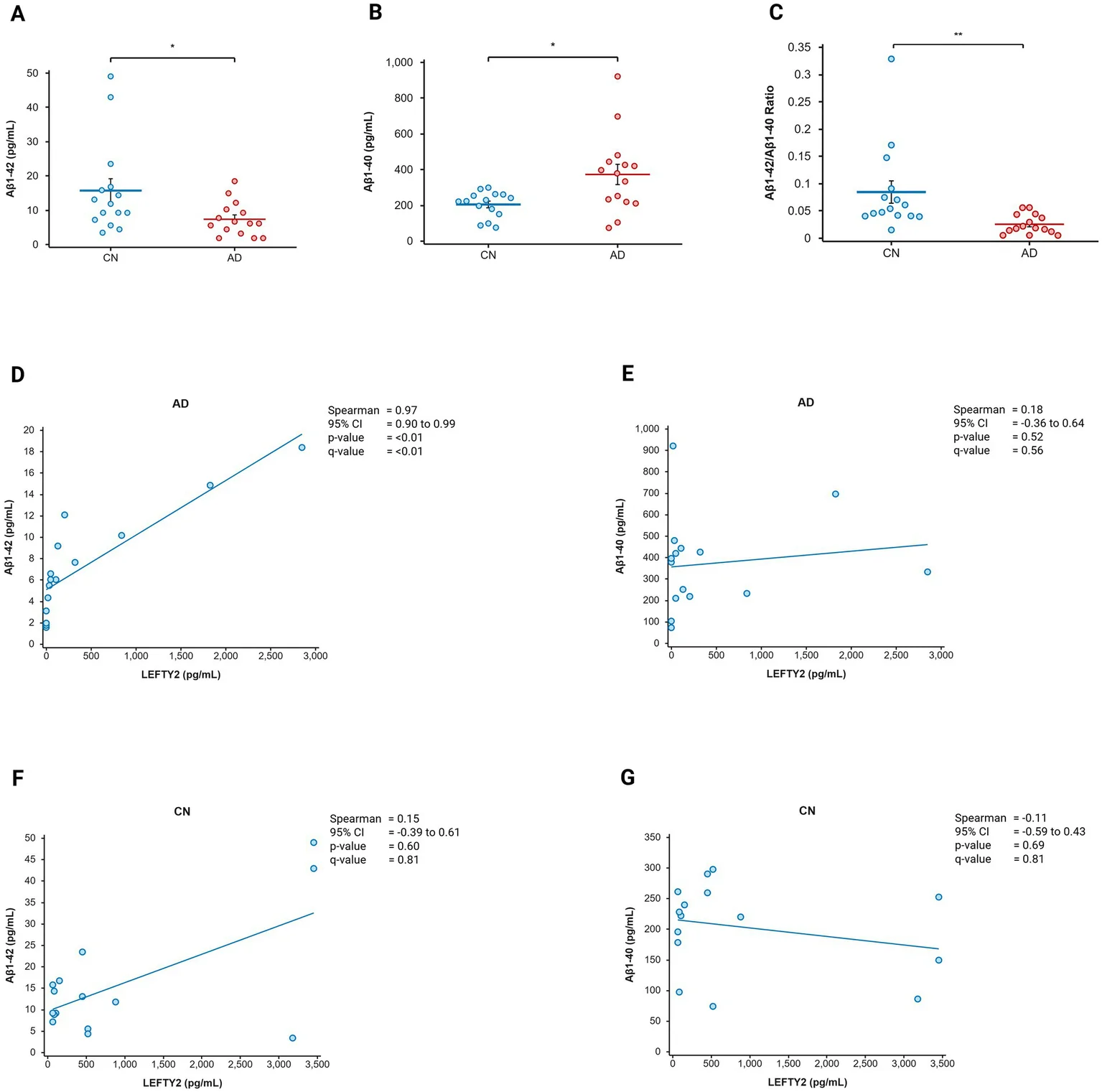
Serum Aβ biomarker levels and Spearman correlations with LEFTY2. Dot plots showing serum concentrations of **(A)** Aβ1-42, **(B)** Aβ1-40, and serum ratios of **(C)** Aβ1-42/Aβ1-40 in cognitively normal (CN) controls and Alzheimer’s disease (AD) patients. Serum Aβ1-40 levels were significantly elevated in AD patients, whereas Aβ1-42 was significantly elevated in CN. The Aβ1-42/Aβ1-40 ratio was significantly reduced in AD patients compared with CN. Each data point represents the mean of duplicate measurements from an individual subject, while horizontal lines indicate group means ± SEM. Group comparisons were analyzed using the Mann–Whitney U test. Scatter plots showing Spearman correlations between LEFTY2 and **(D)** Aβ1-42 and **(E)** Aβ1-40 in AD patients. A significant strong positive correlation between LEFTY2 and Aβ1-42 was observed in AD patients, whereas correlations with Aβ1-40 was not significant. Scatter plots showing Spearman correlations between LEFTY2 and **(F)** Aβ1-42 and **(G)** Aβ1-40 in CN. No significant correlations were observed in CN. Linear regression lines are shown for visualization only. Spearman correlation coefficients (ρ), 95% confidence interval (CI), corresponding *p*-values (calculated using t-distribution approximation), and q-values are indicated within each correlation panel. *n* = 15 for each group.

To further investigate the relationship between circulating LEFTY2 and amyloid-related biomarkers, Spearman correlation analyses were performed. In the AD cohort, serum LEFTY2 levels showed a positive correlation with Aβ1-42 (Spearman = 0.97; 95% CI: 0.90 to 0.99; *p* < 0.01; q < 0.01; Figure 2D), whereas no significant correlation was observed with Aβ1-40 (Figure 2E). In cognitively normal controls, LEFTY2 did not exhibit significant associations with either Aβ1-42 or Aβ1-40 (Figures 2F,G).

Overall, these results suggest a potential association between circulating LEFTY2 and Aβ1-42 in the AD cohort, whereas no corresponding association was observed with Aβ1-40.

### 3.3 LEFTY2 shows a selective association with total tau in cognitively normal controls

Serum pTau181 was significantly elevated in AD patients, whereas pTau181 levels in cognitively normal control were consistently below the assay limit of detection (cognitively normal = 2.00 ± 0.00 pg./mL, 95% CI: 2.00 to 2.00; AD = 23.32 ± 5.31 pg./mL, 95% CI: 12.91 to 33.73; *p* < 0.01; q < 0.01; Figure 3A), supporting the role of tau hyperphosphorylation as a disease-associated hallmark of AD. Similarly, serum pTau231 was significantly higher in the AD group (cognitively normal = 0.02 ± 0.00 pg./mL, 95% CI: 0.02 to 0.02; AD = 0.19 ± 0.07 pg./mL, 95% CI: 0.04 to 0.33; *p* < 0.01; q < 0.01; Supplementary Figure 4); however, due to extremely low concentrations near the limit of detection in both groups, pTau231 was not included in subsequent correlation analysis. In contrast, total tau did not differ significantly between groups (Figure 3B). The pTau181/total tau ratio was markedly elevated in AD patients compared with cognitively normal controls (cognitively normal = 0.03 ± 0.006, 95% CI: 0.02 to 0.04; AD = 0.39 ± 0.12, 95% CI: 0.16 to 0.61; *p* < 0.01; q < 0.01; Figure 3C), further supporting increased pathological tau phosphorylation relative to total tau burden in AD (Zielinska and Gorodkiewicz, 2026).

**Figure 3:**
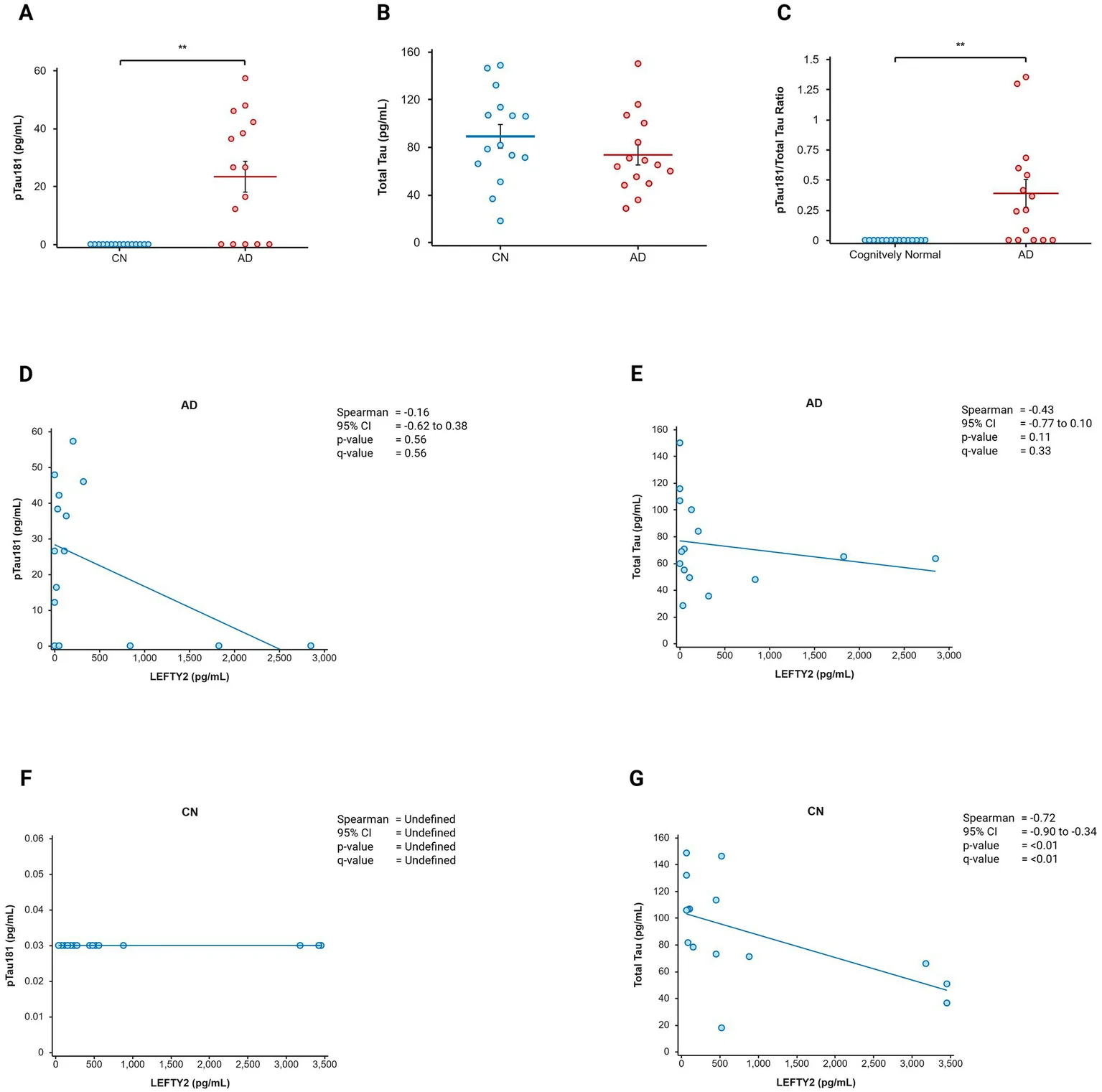
Serum tau-related biomarker levels and Spearman correlations with LEFTY2. Dot plots showing serum concentrations of **(A)** pTau181 and **(B)** total tau, as well as the ratios of **(C)** pTau181/total tau in cognitively normal (CN) controls and patients with Alzheimer’s disease (AD). Serum pTau181 levels were significantly elevated in AD patients, whereas total tau levels did not differ significantly between groups. The pTau181/total tau ratios were significantly increased in AD patients compared with CN. Each data point represents the mean of duplicate measurements from an individual subject, while horizontal lines indicate group means ± SEM. Group comparisons were analyzed using unpaired Student’s t-test for total tau and the Mann–Whitney U test for others. Scatter plots showing Spearman correlations between LEFTY2 and **(D)** pTau181 and **(E)** total tau in AD patients. No significant correlations were observed in AD patients. Scatter plots showing Spearman correlations between LEFTY2 and **(F)** pTau181, and **(G)** total tau in CN. A significant strong negative correlation between LEFTY2 and total tau was observed in CN, whereas correlation with pTau181 was weak and non-significant. Spearman correlation coefficients (ρ), 95% confidence interval (CI), corresponding *p*-values (calculated using t-distribution approximation), and q-values are indicated within each correlation panel. *n* = 15 for each group.

Correlation analyses revealed that none of the assessed tau-related biomarkers showed significant correlations with circulating LEFTY2 in AD patients (Figures 3D,E). In contrast, circulating LEFTY2 demonstrated a significant negative correlation with total tau in cognitively normal controls (Spearman = −0.72; 95% CI: −0.90 to −0.34; *p* < 0.01; q < 0.01; Figure 3G), whereas pTau181 was largely undetectable, resulting in a floor effect and precluding reliable correlation analysis with LEFTY2 (Figure 3F).

Collectively, these findings suggest that serum LEFTY2 is negatively associated with total tau under physiological conditions, whereas no significant correlations with tau biomarkers were observed in AD patients.

### 3.4 LEFTY2 shows a selective association with TGF-β1 in cognitively normal controls

In a previous AD cell model study, recombinant LEFTY2 treatment increased TREM2 expression and reduced MIF levels (Wuli et al., 2025). To determine whether similar patterns are observed in peripheral blood, we measured serum MIF and TREM2. Given that LEFTY2 is a member of the TGF-β superfamily, we additionally measured serum TGF-β1 to explore its potential relationship with LEFTY2 (Chen and Shen, 2004).

In the present cohort, serum MIF and TREM2 levels showed no significant differences between groups (Figures 4A,B). Furthermore, serum LEFTY2 levels were not significantly correlated with either MIF or TREM2 in AD (Figures 4D,E) or cognitively normal controls (Figures 4G,H).

**Figure 4:**
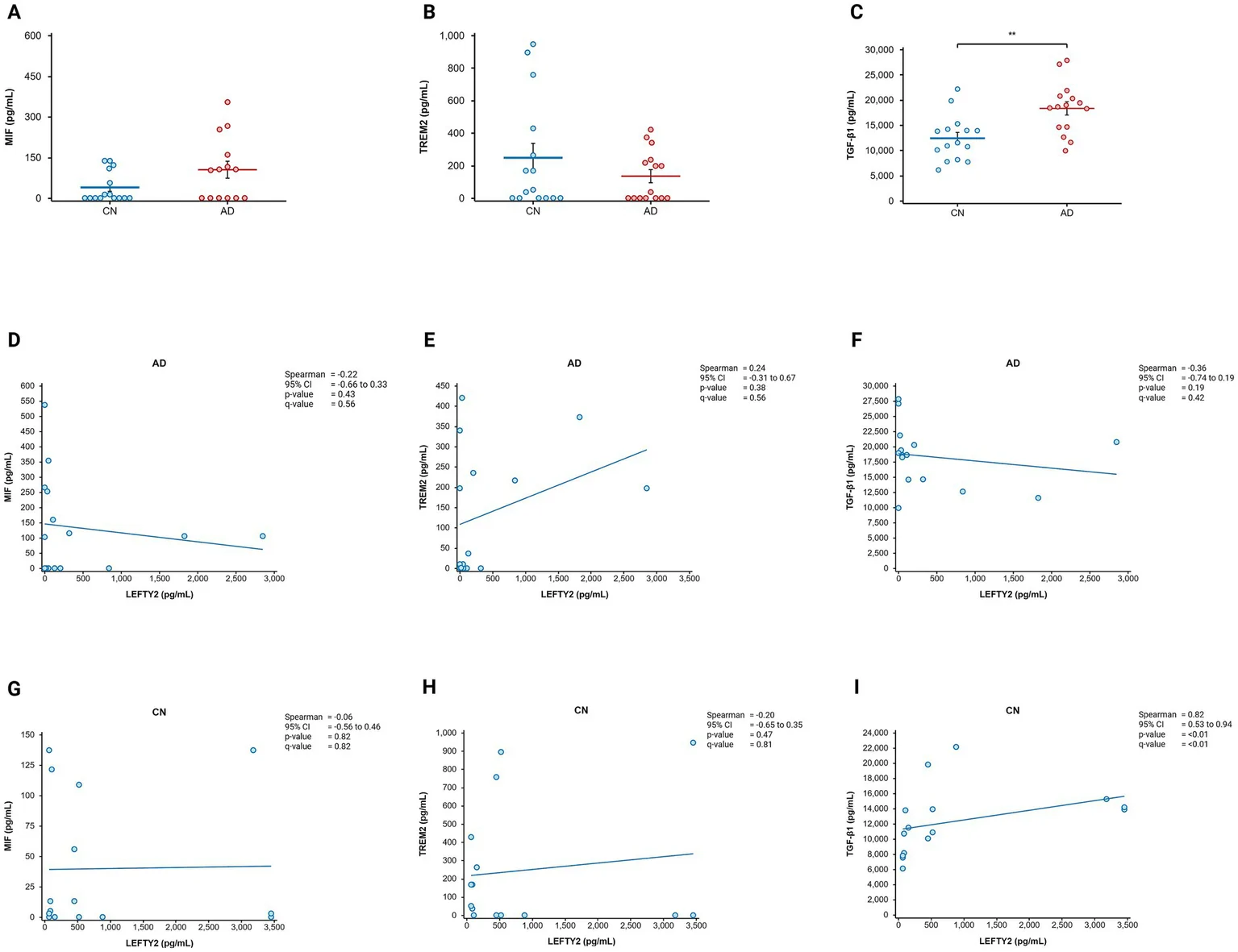
Serum inflammatory-related biomarker levels and Spearman correlations with LEFTY2. Scatter plots showing serum concentrations of **(A)** MIF, **(B)** TREM2, and **(C)** TGF-β1 in cognitively normal (CN) controls and Alzheimer’s disease (AD) patients. Each data point represents the mean of duplicate measurements from an individual subject, while horizontal lines indicate group means ± SEM. TGF-β1 was significantly elevated in AD patients, whereas no significant differences in serum MIF or TREM2 levels were observed between groups. Group comparisons were analyzed using the Mann–Whitney U test. Scatter plots showing Spearman correlations between LEFTY2 and **(D)** MIF, **(E)** TREM2, and **(F)** TGF-β1 in AD patients. No significant correlations were observed in AD patients. Scatter plots showing Spearman correlations between LEFTY2 and **(G)** MIF, **(H)** TREM2, and **(I)** TGF-β1 in CN. A significant positive correlation between LEFTY2 and TGF-β1 was observed in cognitively normal controls, whereas correlations with MIF and TREM2 were weak and non-significant. Spearman correlation coefficients (ρ), 95% confidence interval (CI), corresponding *p*-values (calculated using t-distribution approximation), and q-values are indicated within each correlation panel. *n* = 15 for each group.

In contrast, serum TGF-β1 was significantly elevated in the AD group relative to cognitively normal controls (cognitively normal = 12390.79 ± 1155.88 pg./mL, 95% CI: 10125.27 to 14656.30; AD = 18312.50 ± 1322.77 pg./mL, 95% CI: 15719.86 to 20905.13; *p* < 0.01; q < 0.01; Figure 4C). Serum LEFTY2 levels were positively correlated with TGF-β1 levels in cognitively normal controls (Spearman = 0.82; 95% CI: 0.53 to 0.94; *p* < 0.01; q < 0.01; Figure 4I). This association was not observed in AD patients (Figure 4F).

Collectively, these data indicate that serum LEFTY2 is positively associated with TGF-β1 in cognitively normal controls, whereas no significant associations with MIF or TREM2 were observed in either group.

### 3.5 NF-L is elevated in AD with limited association to LEFTY2

NF-L is a widely used blood-based marker of axonal injury and neurodegeneration and has been reported to increase in AD and other neurodegenerative conditions (Buonocore et al., 2025). We therefore measured serum NF-L to determine whether circulating LEFTY2 is associated with neurodegeneration-related changes in AD.

Serum NF-L, was significantly elevated in AD patients compared with cognitively normal controls (cognitively normal = 19.50 ± 6.64 pg./mL, 95% CI: 6.48 to 32.52; AD = 64.14 ± 14.02 pg./mL, 95% CI: 36.67 to 91.61; *p* < 0.01; q = 0.02; Figures 5A,B), suggesting an increased neurodegeneration in AD. To further evaluate the relationship between LEFTY2 and neurodegeneration, correlation analyses were performed. Serum LEFTY2 demonstrated a positive correlation with NF-L in AD patients (Spearman = 0.56; 95% CI: 0.06 to 0.83; *p* = 0.03); however, this association did not retain statistical significance following Benjamini–Hochberg FDR adjustment (q = 0.13; Figure 5C). No significant correlation was observed in the cognitively normal control group (Figure 5D).

**Figure 5:**
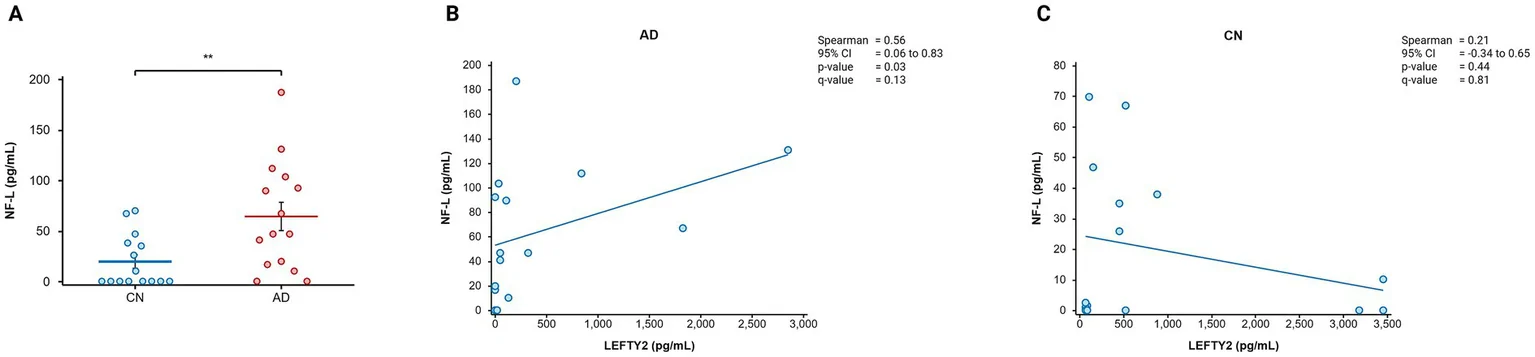
Serum NF-L levels and Spearman correlations with LEFTY2. **(A)** Scatter plots showing serum NF-L concentrations in cognitively normal (CN) controls and Alzheimer’s disease (AD) patients. Each data point represents the mean of duplicate measurements from an individual subject, while horizontal lines indicate group means ± SEM. Serum NF-L levels were significantly elevated in AD patients compared with cognitively normal controls. Group comparisons were analyzed using the Mann–Whitney U test. Scatter plots showing Spearman correlations between LEFTY2 and NF-L in **(B)** AD patients and **(C)** CN. No significant correlations were observed in either cohort. Spearman correlation coefficients (ρ), 95% confidence interval (CI), corresponding *p*-values (calculated using t-distribution approximation), and q-values are indicated within each correlation panel. *n* = 15 for each group.

Overall, these results suggest a lack of robust association between serum LEFTY2 and NF-L in this cohort.

## 4 Discussion

In this exploratory study, we observed disease-dependent associations between LEFTY2 and blood biomarkers (Figure 6). Serum LEFTY2 levels is significantly lower in patients with Alzheimer’s disease (AD), accompanied by an AD-specific positive correlation with serum Aβ1-42 (Spearman = 0.97). AD patients also showed lower serum Aβ1-42, higher Aβ1-40, and a reduced Aβ1-42/Aβ1-40 ratio compared with cognitively normal controls, a pattern directionally consistent with established AD-related amyloid alterations (Perez-Grijalba, 2019; Lopez, 2020).

**Figure 6:**
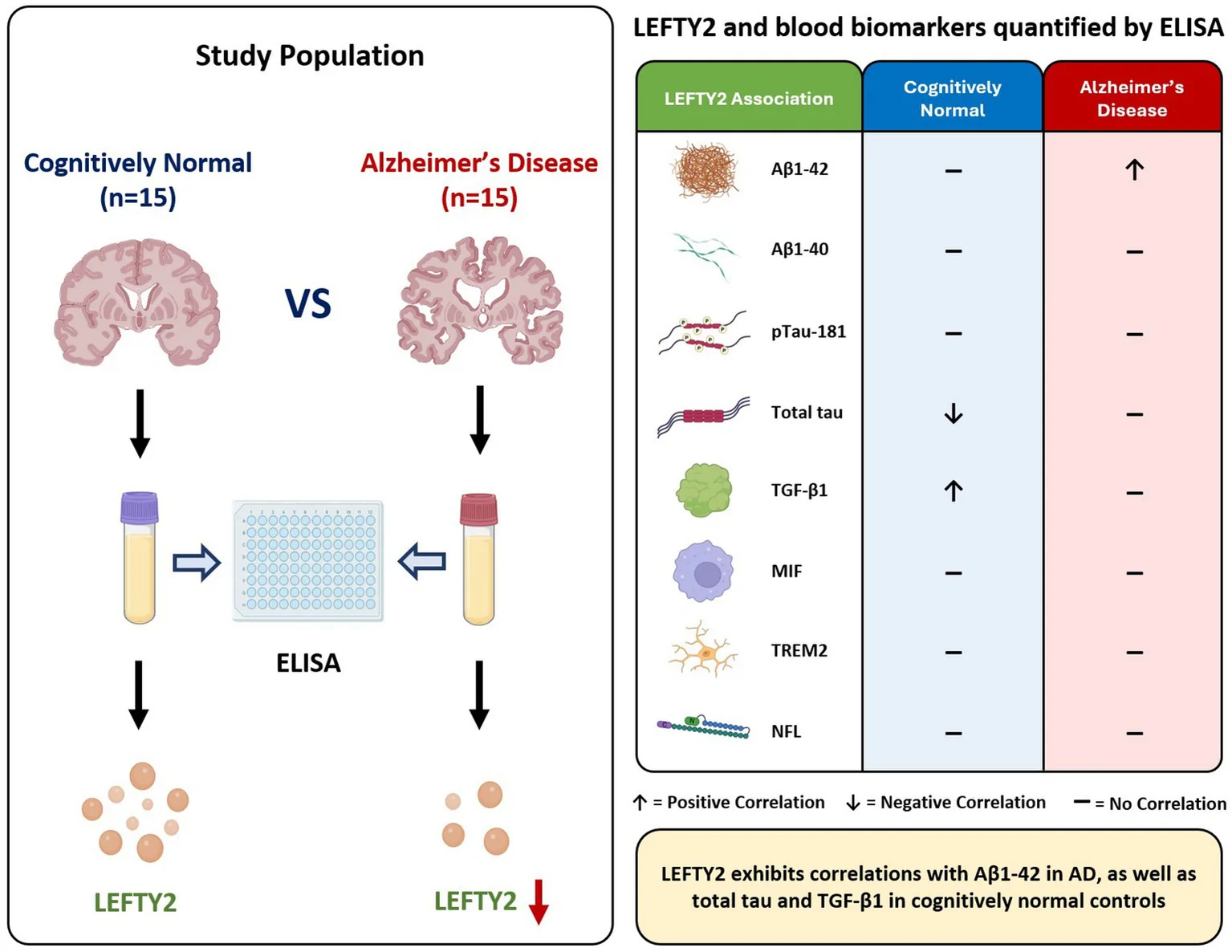
Correlations between serum LEFTY2 and blood biomarkers in cognitively normal (CN) controls and Alzheimer’s disease (AD) patients. In CN, LEFTY2 positively correlates with TGF-β1 and negatively correlates with total tau, whereas in AD, it positively correlates with Aβ1-42. No significant correlations were observed for Aβ1-40, pTau-181, MIF, TREM2, or NF-L in either cohort.

The association between LEFTY2 and Aβ1-42 observed exclusively in the AD cohort suggests a potential disease-dependent relationship between these biomarkers. However, the cross-sectional and correlational design precludes conclusions regarding causality or shared upstream regulation. Serum Aβ measurements are influenced by pre-analytical factors, matrix effects, peripheral sources, and assay platforms and may not directly reflect CNS amyloid burden (Hansson et al., 2022; Verberk et al., 2022). Thus, the present serum Aβ findings should be interpreted as exploratory rather than as a direct measure of brain amyloid burden.

Associations between LEFTY2 and tau-related biomarkers were limited. LEFTY2 was negatively correlated with total tau exclusively in cognitively normal controls, suggesting a potential relationship with tau regulation that may be altered in AD. In contrast, despite elevated serum pTau181 in AD patients, no significant association with LEFTY2 was observed. Interpretation of pTau181 was limited by the low or undetectable concentrations in cognitively normal controls, highlighting the analytical challenges of measuring low-abundance phosphorylated tau species in serum (Karikari et al., 2022; Zetterberg et al., 2013).

Recent studies have identified pTau217 as one of the most informative blood-based biomarkers for AD, with strong associated with amyloid and tau pathology (Yang et al., 2026; Ashton et al., 2024). Although pTau217 was not assessed in the present study, future investigation of its relationship with serum LEFTY2 may provide further insight into the association between LEFTY2 and AD-related tau pathology.

Serum MIF and TREM2 levels showed no group-level differences or significant correlations with serum LEFTY2. However, the absence of serum correlations for MIF and TREM2 should not be interpreted as an absence of biological involvement in neuroinflammatory or microglial processes in AD. Peripheral concentrations of cytokines and microglial-associated proteins may not directly reflect CNS-specific inflammatory activity, partly because of compartmentalization between the peripheral circulation and CNS. Consequently, these serum findings warrant further investigation in cerebrospinal fluid (CSF), brain tissue, and mechanistic models to clarify their relationship with CNS inflammatory processes.

TGF-β1 was significantly higher in AD patients than in cognitively normal controls. Among the inflammatory and neurodegeneration-related markers assessed, only TGF-β1 showed a significant positive correlation with LEFTY2 in cognitively normal controls. Given that LEFTY2 is involved in the regulation of TGF-β superfamily signaling, this association potentially reflecting a classical negative feedback mechanism where physiological TGF-β1 expression induces LEFTY2 to maintain pathway signaling balance (Chen and Shen, 2004). Notably, despite the overall elevation of TGF-β1 in AD, its association with LEFTY2 was no longer observed, suggesting that increased TGF-β1 levels in AD were not in parallel with LEFTY2. Whether this disease-dependent difference reflects altered TGF-β-related regulation or other AD-associated processes requires further investigation in larger cohorts and mechanistic models.

Serum NF-L was significantly elevated in AD patients, suggesting an increased axonal injury and neurodegeneration. However, NF-L is a general marker of axonal damage rather than an AD-specific biomarker which limits its diagnostic specificity for AD (Pan et al., 2025). Although LEFTY2 showed a nominal positive correlation with NF-L in AD, this association did not remain significant after FDR correction, providing limited evidence for a robust relationship between the two biomarkers in this cohort. Given the exploratory sample size, larger studies are needed to determine whether this nominal association is reproducible.

Several limitations should be considered when interpreting these findings. The modest sample size (*n* = 15 per group) and cross-sectional design limit statistical power and preclude causal interpretation of the observed associations. Moreover, the relatively large number of biomarker comparisons and correlation analyses increases the probability of false-positive findings due to multiple testing. Although the Benjamini–Hochberg FDR procedure was applied to reduce this risk, residual uncertainty remains given the exploratory nature and modest sample size of the present study. In addition, the modest sample size may increase the uncertainty and potential inflation of the observed correlation estimates. Peripheral serum concentrations may not directly reflect CNS pathology. Therefore, these findings should be considered exploratory and require validation in larger, independent cohorts.

Because the present study was exploratory and not specifically designed to evaluate diagnostic performance, sensitivity, specificity, and receiver operating characteristic (ROC) analyses were not performed. Accordingly, the present findings should be regarded as hypothesis-generating rather than evidence of diagnostic validity. The cross-sectional and correlational design also does not establish the direction of the observed associations; specifically, it cannot determine whether changes in LEFTY2 influence amyloid-related processes, whether amyloid-related changes influence circulating LEFTY2, or whether both are driven by shared upstream biological factors.

Future prospective studies with longitudinal follow-up and independent validation cohorts will therefore be required to determine the clinical utility of LEFTY2 as a potential blood-based biomarker. Such studies should evaluate serum LEFTY2 across the AD continuum, including subjective cognitive decline (SCD), mild cognitive impairment (MCI) and biomarker-defined preclinical AD (Frisoni et al., 2022; Antonioni, 2026). Integration with ultrasensitive blood biomarkers such as pTau217, CSF measurements, and amyloid- and tau-PET imaging may further help determine how circulating LEFTY2 relates to disease progression and central AD pathology.

Overall, serum LEFTY2 showed a disease-dependent association with Aβ1-42, whereas its relationships with tau-related, neurodegenerative, and inflammatory biomarkers were more limited or context-dependent. These findings support further investigation of the potential relationship between LEFTY2 and amyloid-related processes in AD.

## 5 Conclusion

In summary, this exploratory pilot study identifies a significant reduction in serum LEFTY2 levels in patients with Alzheimer’s disease, accompanied by distinct disease-dependent biomarker associations, specifically with Aβ1-42 in AD, as well as total tau and TGF-β1 in cognitively normal controls. While cross-sectional and observational in nature, these findings suggest that circulating LEFTY2 exhibits disease-dependent relationships with selected AD-related biomarkers. Future longitudinal investigations in larger, well-characterized cohorts, incorporating paired central biomarkers, are warranted to clarify the biological significance and potential biomarker utility of LEFTY2 across the AD continuum.

## Data Availability

The original contributions presented in the study are included in the article/Supplementary material, further inquiries can be directed to the corresponding authors.
