# Supplementary material for: Reduced serum LEFTY2 and disease-dependent biomarker associations in Alzheimer’s disease: an exploratory study
Source: Front Aging Neurosci. 2026 Oct 6;18:1955495. doi: 10.3389/fnagi.2026.1955495 (PMC13680440; doi:10.3389/fnagi.2026.1955495)
Supplement: Supplementary file 1 [file Data_Sheet_1.docx]

Supplementary Material

# 1 Supplementary Figures

**Supplementary Figure 1** Sensitivity analysis of serum biomarker levels after outlier removal. Scatter plots displaying sensitivity analyses for serum (A) LEFTY2, (B) Aβ1-42, and (C) Aβ1-40 levels between cognitively normal (CN) controls and Alzheimer's Disease (AD) patients following the exclusion of statistical outliers identified via the ROUT method. Each data point represents the mean of duplicate measurements from an individual subject, while horizontal lines indicate group means ± SEM. Consistent with primary findings, group differences remained statistically significant across all three biomarkers after outlier removal. Group comparisons were analysed using the Mann-Whitney U test. n = 12 for each group.

**Supplementary Figure 2** Sensitivity analysis of correlations between serum LEFTY2 and blood-biomarkers in the AD patients after outlier removal. Scatter plots showing Spearman correlations between LEFTY2 and **(A**) Aβ1-42, **(B)** Aβ1-40 **(C)** pTau231, **(D)** pTau181, **(E)** total tau, **(F)** MIF, **(G)** TREM2, **(H)** TGF-β1, and **(I)** NF-L. Listwise deletion was applied to exclude participants identified as LEFTY2 outliers along with their corresponding paired biomarker measurements. Consistent with the primary findings, outlier removal did not alter the significance of the correlation results. Spearman correlation coefficients (ρ), 95% confidence interval (CI), corresponding p-values (calculated using t-distribution approximation), and q-values are indicated within each correlation panel. n = 12 for each group.

**Supplementary Figure 3** Sensitivity analysis of correlations between serum LEFTY2 and blood-biomarkers in the cognitively normal (CN) controls after outlier removal. Scatter plots showing Spearman correlations between LEFTY2 and **(A**) Aβ1-42, **(B)** Aβ1-40 **(C)** pTau231, **(D)** pTau181, **(E)** total tau, **(F)** MIF, **(G)** TREM2, **(H)** TGF-β1, and **(I)** NF-L. Listwise deletion was applied to exclude participants identified as LEFTY2 outliers along with their corresponding paired biomarker measurements. Consistent with the primary findings, outlier removal did not alter the significance of the correlation results. Spearman correlation coefficients (ρ), 95% confidence interval (CI), corresponding p-values (calculated using t-distribution approximation), and q-values are indicated within each correlation panel. n = 12 for each group.

**Supplementary Figure 4** Serum pTau231 levels. Scatter plots showing serum pTau231 concentrations in cognitively normal (CN) controls and Alzheimer’s disease (AD) patients. Each data point represents the mean of duplicate measurements from an individual subject, while horizontal lines indicate group means ± SEM. Serum pTau231 levels were significantly elevated in AD patients compared with CN; however, due to extremely low concentrations near the limit of detection in both groups, pTau231 was not included in subsequent correlation analysis. Group comparisons were analysed using the Mann-Whitney U test.

**Supplementary Figure 5** Technical replicates for ELISA assays**.** Plots show technical replicates for **(A)** LEFTY2, **(B**) Aβ1-42, **(C)** Aβ1-40 **(D)** pTau231, **(E)** pTau181, **(F)** total tau, **(G)** MIF, **(H)** TREM2, **(I)** TGF-β1, and **(J)** NF-L. Linear regression lines and R^2^ values are shown in each panel. All assays demonstrated excellent linearity (R^2^ > 0.995).
